# Supplementary material for: Control of cascading failures using protective measures
Source: Sci Rep. 2024 Jun 23;14:14444. doi: 10.1038/s41598-024-65379-5 (PMC11194283; doi:10.1038/s41598-024-65379-5)
Supplement: Supplementary file 1 — Supplementary Information. [file 41598_2024_65379_MOESM1_ESM.pdf]

Supplementary Materials for

Control of cascading failures using protective measures

Davood Fazli, Mozhgan Khanjanianpak, Nahid Azimi-Tafreshi

1 The effect of structure on the dynamics

In the results of the model on the random network, we observed that in a certain range of parameters α and σ, the density of protected nodes shows the oscillatory behavior. Also a bistable region with a discontinuous phase transition appears in some range of the parameters. Here we aim to show the results of the model on the small-world and the scale-free networks.

Let us consider the model on a small-world network, produced following the Watts-Strogatz algorithm [1]. We start with a ring having N nodes and node degree $k = 2n$, such that each node is connected to its n nearest neighbors on each side. Moving clockwise, for every node we select randomly an edge that connects that node to one of its neighbors, and rewire it with probability p. We continue this process until each edge in the original ring has been considered once. The parameter p measures the degree of disorder or randomness of the resulting network.

We consider the dynamics of the model on a small-world network with $N = 10^4$ and $n = 5$. Other parameters set as $\beta = 0.3$, $m = 0.2$ and $c = 1$. Figure S1 shows the stationary behavior of the density of active and protected nodes for $p = 0.02$. For even small values of p, the small-world effect emerges which is the root of oscillations. With increasing p, the amplitude of oscillations grows (see Fig. S2). However for large value of network randomness ($p = 0.9$), we also observe a bistable region in Fig. S3. We hence conjecture that a necessary condition for the discontinuous transitions and the bistable region is the existence of long loops in the underlying networks.

Figure S4 shows the results for a scale-free network with degree distribution $P(k) \propto k^{-\gamma}$, such that γ is set to 2.1. As we can see the behaviour is qualitatively similar to the random networks.

References

[1] D. J. watts, D. J. & Strogatz, S. H. Collective dynamics of small-world networks, *Nature* **393**, 440 (1998).

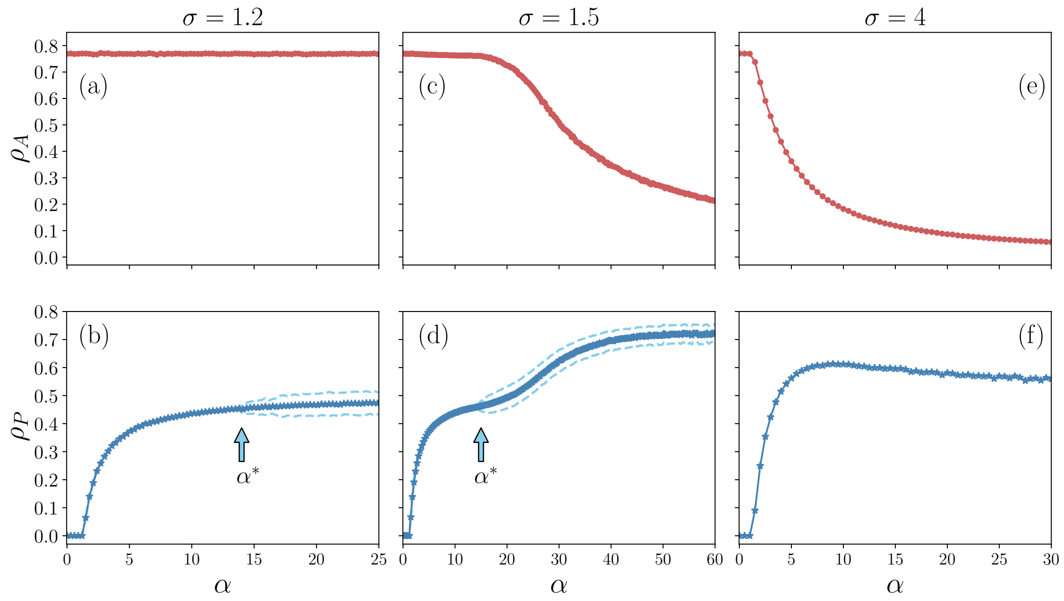

Figure S1: Stationary values of the cascade size (red dots) and the fraction of protected nodes (blue stars) as a function of α on a small-world network with rewiring probability $p = 0.02$. The value of σ increases from left to right. Bifurcation point α^* is shown in the figure. Dashed lines show the upper and lower turning points of stable cycles. The results are averaged over 500 realizations.

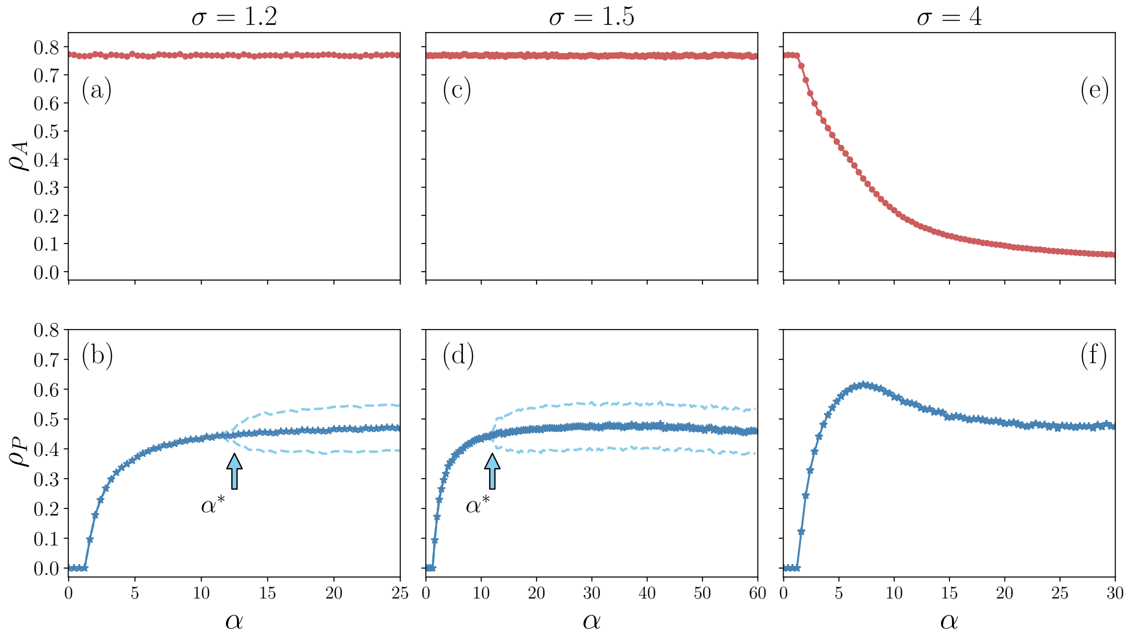

Figure S2: Stationary values of the cascade size (red dots) and the fraction of protected nodes (blue stars) as a function of α on a small-world network with rewiring probability $p = 0.5$. The value of σ increases from left to right. Bifurcation point α^* is shown in the figure. Dashed lines show the upper and lower turning points of stable cycles. The results are averaged over 500 realizations.

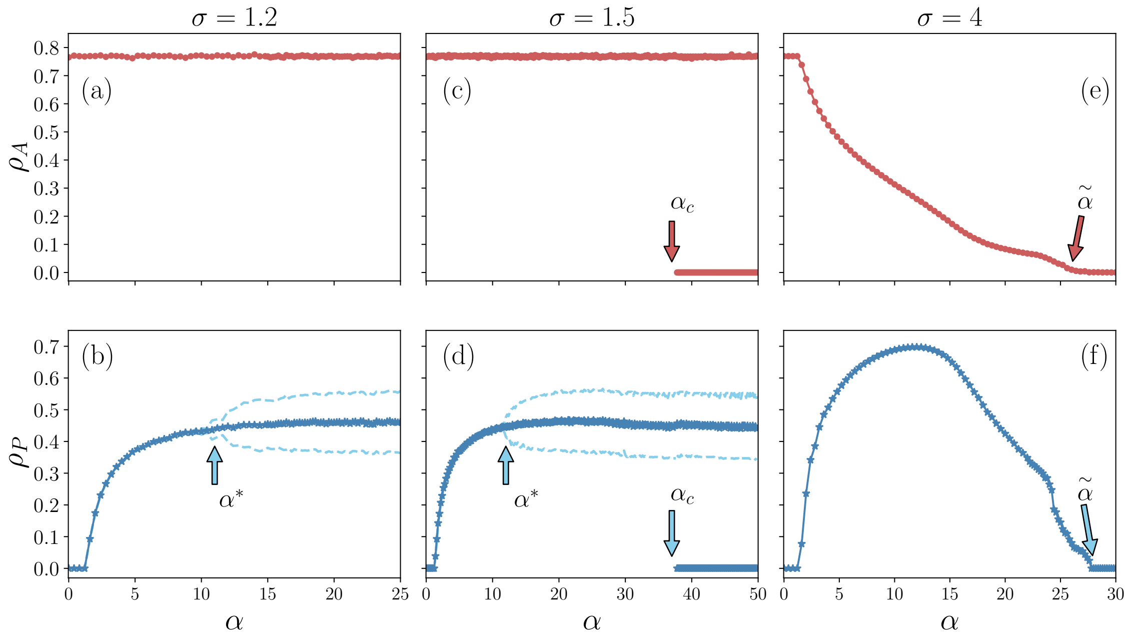

Figure S3: Stationary values of the cascade size (red dots) and the fraction of protected nodes (blue stars) as a function of α on a small-world network with rewiring probability $p = 0.9$. The value of σ increases from left to right. Bifurcation point α^*, bistability point α_c, and absorbing point $\tilde{\alpha}$ are shown in the figure. Dashed lines show the upper and lower turning points of stable cycles. The results are averaged over 500 realizations.

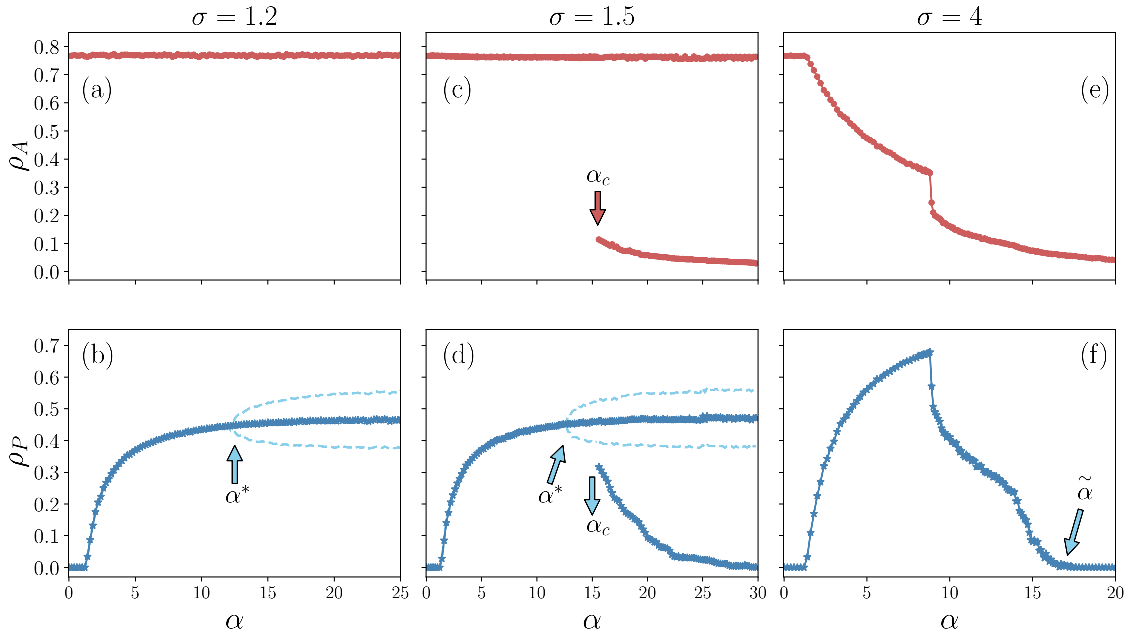

Figure S4: Stationary values of the cascade size (red dots) and the fraction of protected nodes (blue stars) as a function of α on a scale-free network with degree distribution $P(k) \propto k^{-2.1}$. The value of σ increases from left to right. Bifurcation point α^*, bistability point α_c, and absorbing point $\tilde{\alpha}$ are shown in the figure. Dashed lines show the upper and lower turning points of stable cycles. The results are averaged over 500 realizations.
